# Supplementary material for: NipahVR: a resource of multi-targeted putative therapeutics and epitopes for the Nipah virus
Source: Database (Oxford). 2020 Feb 24;2020:baz159. doi: 10.1093/database/baz159 (PMC7036594; doi:10.1093/database/baz159)
Supplement: Manuscript_color_change_baz159 [file manuscript_color_change_baz159.docx]

**NipahVR: multi-targeted putative therapeutics and epitome resource for the nipah virus**

**NipahVR: a resource of multi-targeted putative therapeutics and epitopes for the nipah virus**

Amit Kumar Gupta, Archit Kumar, Akanksha Rajput, Karambir Kaur, Showkat Ahmed Dar, Anamika Thakur, Kirti Megha and Manoj Kumar*

Virology Unit and Bioinformatics Centre, Institute of Microbial Technology, Council of Scientific and Industrial Research (CSIR), Sector 39-A, Chandigarh-160036, India

* To whom correspondence should be addressed. Tel, 91-172-6665453; Fax, 91-172-2690585; 91-172-2690632; Email, [manojk@imtech.res.in](mailto:manojk@imtech.res.in)

**Abstract**

Nipah virus (NiV) is an emerging and priority pathogen from the *Paramyxoviridae* family with a high fatality rate. It causes various diseases such as respiratory ailments, encephalitis, etc., and poses a great threat to humans and livestock. Despite all efforts, there is no approved vaccine or therapeutics is available for Nipah infection. Despite various efforts, there is no approved antiviral treatment available. Therefore, to expedite and assist the research, we have developed an integrative resource NipahVR (<http://bioinfo.imtech.res.in/manojk/nipahvr/>) for the multi-targeted putative therapeutics and epitome epitopes for NiV. It is structured into different sections, i.e., genomes, codon usage, phylogenomics, molecular diagnostic primers, therapeutics (siRNAs, sgRNAs, miRNAs) and vaccine epitopes (B-cell epitopes, CTL epitopes, MHC-I and -II binders). Most decisively, potentially efficient therapeutic regimens targeting different NiV proteins and genes were anticipated and projected. We hope this computational resource would be helpful in developing combating strategies against this deadly pathogen.

**Database URL:** <http://bioinfo.imtech.res.in/manojk/nipahvr/>

**Keywords:** Nipah virus, Therapeutics, Vaccine epitope, diagnostics, siRNAs, miRNAs, sgRNAs

**Introduction**

Nipah virus (NiV) is a highly pathogenic virus closely related to the Hendra virus (HeV) from the genus *Henipavirus* of the family *Paramyxoviridae*. NiV is an enveloped single-stranded RNA (ssRNA), a negative-sense virus of size ~18,250 nucleotides. It encodes nine proteins namely nucleoprotein (N), four proteins encoded by P gene (phosphoprotein (P), W, V, C protein), matrix (M) protein, fusion (F) glycoprotein, attachment glycoproteins (G), and a large polymerase (L) protein (1-3).

Different NiV proteins play a cardinal role in viral infection and disease manifestation. Viral G protein (attachment protein) first binds to the Ephrin-B2 or B3 cellular receptors found on the neuron, smooth muscles, capillaries, and arterial endothelial cells. G protein provides attachment to the host cell surface, which triggers the fusion by the F protein(4,5). Subsequently, F protein, a 546 amino acid (aa) long type-I transmembrane protein, which mediates fusion of virus and host cell membranes and mediates cell entry(6). Then, viral RNA content gets synthesized and translated into the proteins. P gene encodes four gene products. A structural P protein (709 aa) essential for genome replication encoded by unedited mRNA and localized in the cytoplasm. The three additional non-structural proteins (V, W, and C) contribute towards the evasion of the innate immune response through inhibiting the different signaling pathways and are crucial for the viral infection. V and W proteins are produced by RNA editing and localized in cytoplasm and nucleus, respectively, and the second open reading frame (ORF) generates C protein. M protein (352 aa) has a crucial role in viral budding. It also provides firmness to virion through interacting with envelope and F protein. M protein is also known to hijack cellular pathways and machines to facilitate nuclear localization. N protein (532 aa) is mainly responsible for the viral genome encapsidating. The largest NiV protein L retains all the enzymatic functions like genome replication and transcription for the viral RNA synthesis(1-3).

NiV is an emerging zoonotic virus classified as a category C priority pathogen and Biosafety Level-4 (BSL-4) agent that signify a rolling threat to humans and animals worldwide(7,8). It was originated and first isolated from the village "Sungai Nipah"(7,8). It is an etiological agent of diverse diseases such as encephalitis, respiratory illness, and fever(9,10). Epidemiologically, NiV transmission occurs mainly through infected animals (bats, pigs, etc.), contaminated food consumption(11), and may spread through person to person(12). Flying foxes (fruit bats) from the genus *Pteropus* are known as a natural reservoir and host of the NiV(13). Among all *Pteropus* species, *P. giganteus* (Indian flying fox) mainly distributed in south Asian regions like Bangladesh, India, and Pakistan.(14,15). Other species were also found in different parts of Southeast Asia like *P. vampyrus* and *P. hypomelanus* in Malaysia, *P. lylei* in Thailand and Cambodia(16,17).

Up to now, various sporadic outbreaks were reported from the different countries, mainly from South Asia, i.e., India, Bangladesh, Malaysia, and Singapore, since the first incidence of Malaysia in 1998 with the high mortality rate between 40-75% depending on clinical manifestations(3,7,8,18,19). These are mainly endemic in India and Bangladesh (7,8,20,21). In India, the first outbreak was reported from the Siliguri, West Bengal, in 2001 with high fatalities due to NiV encephalitis (18,22). In this, the involvement of pigs as an infection mediator is not observed, and direct person-to-person transmission was reported that signify high risk to public health (22). Later in 2007, another outbreak was reported from West Bengal with 100% mortality (23). Very recently, in May 2018, the first NiV outbreak occurred in southern India in Kozhikode and Malappuram districts of Kerala. Several deaths were reported due to the unavailability of a practical solution, which is of concern to India and the world (7,8,24,25). Moreover, a study also describes the presence of NiV RNA in *Pteropus giganteus* in different Indian states signifying it as a natural reservoir in India(15). However, more surveillance studies are necessary to access the NiV outbreak risk among susceptible populations living in different geographical locations(7). Furthermore, several NiV outbreaks were also documented from Bangladesh between 2001 to 2015(7,11,26). These are linked to many deaths due to encephalitis with neurological and respiratory complications(11,26). Moreover, different studies also provide information about NiV origin, evolution, and stability over time (7,8). Like, a study shows the conservation between the isolates from Bangladesh, 2004, and India, 2007 with 99.2% and 99.8%, nucleotide and amino acid similarity, respectively (23). Similarly, another study also provides phylogenetic analysis and conservation of NiV, i.e., between 96-100% (25). Moreover, a recent report by Ravichandran et al. have also found conservancy among NiV proteins (27).

There are also efforts to combat the NiV, and different strategies (vaccines, immunotherapies, antiviral drugs) were tried to eradicate the infection(28). Distinct approaches like subunit vaccine(29), vectored vaccine, live-vectored vaccine(30)mainly utilizing the G and P proteins demonstrated to elicit an immune response(28,31). Likewise, a virus-like particle (VLP)-based vaccine is also shown protection against NiV(32). These experimental vaccines are mainly tested on animal models like a hamster, ferret, cats, pigs, etc.(28,31,32). Moreover, a subunit vaccine for use in horses has been developed based on the HeV G protein (28,31,33). Additionally, the use of monoclonal and polyclonal antibodies also showed success in treating NiV infection in animals (34). However, more studies, i.e., in-vitro as well as in-vivo, will be required before conducting clinical trials.

Likewise, other strategies such as RNA interference (RNAi) through small interfering RNAs (siRNAs) are also used previously to inhibit N and L genes(35). Further, an anti-viral drug, Ribavirin, is also used in infected persons(36) and also tested on animal experiments. However, it did not show good efficiency against the infection (36,37). Very recently, a small-molecule antiviral drug Favipiravir (T-705) have shown the compelling antiviral activity in the hamster model against the henipaviruses (NiV and HeV)(38). Likewise, the potential of natural antiviral agents from the medicinal plants can also be explored to combat viruses(39,40). Furthermore, studies also provide promising small-molecule inhibitors targeting NiV proteins (41,42). Apart from these, there are also some computational efforts to provide solutions in different ways. Like, some studies advocate the computational designing of vaccine epitopes against the specific NiV proteins(43,44). Very recently, we have also developed a quantitative structure-activity relationship (QSAR) based prediction algorithm “*anti-Nipah*” for the identification of effective inhibitors against the NiV(45). The algorithm will predict the antiviral ability of any query compound against the NiV(45).

However, despite determinations, the Nipah Virus study is generally neglected. Currently, there is no Food and Drug Administration (FDA) approved therapeutics or prophylactic vaccine is available to treat NiV diseases in humans, and treatment is only supportive(21,28,31). Simultaneously, a broad range of Nipah hosts, its pathogenesis, and the high fatality rate pose a recurring threat to humanity(46). Therefore, effective vaccines and therapeutics are an inevitable necessity of time(21). In the study, we have made efforts to provide potential therapeutic and vaccine solutions targeting all NiV proteins or genes. The resource "NipahVR" may assist the worldwide scientific community in fighting against this lethal pathogen.

**Materials and methods**

**Data retrieval**

Complete genome sequences of the NiV were searched and collected from the NCBI database. In total, 18 complete or near-complete sequences were obtained utilizing the length filtering criteria of size more than 10kb and provided on the resource (**Table 1**). A facility with category wise (i.e., host, geographical area, and country) search option is applied on the web resource for the ease. Different information such as strain/isolate, host/source, length, country, geographical region, etc. was cataloged. Further, gene and protein sequences of reference NiV genome (NC_002728.1) are used for the downstream analyses, mainly diagnostic primer designing, vaccine epitope prediction, and RNA-based therapeutics (i.e., small interfering RNAs (siRNAs), microRNAs (miRNAs), single guide RNAs (sgRNAs)).

**Codon analysis**

Codon bias analysis of the complete genome sequence is performed to explore relative synonymous codon usage (RSCU) and codon frequency. Further, codon preference and context are analyzed employing the Anaconda program (47).

**Phylogenomics**

We have performed phylogenomic analysis to understand the phylogenetic reconstruction of NiV genomes. In the current study, we have employed complete genomes of 15 NiV that cause outbreaks in various Asian countries like India, Bangladesh, Malaysia, and Singapore from 1998-2018. The genomic information was extracted from various sources like NCBI, ViPR, Viral zone, and research articles (48,49). Further, the Molecular Evolutionary Genetics Analysis (MEGAv7.0) software with a Neighbor-joining method was utilized (50). The evolutionary distance was inferred through the Jukes-Cantor method, with a bootstrap test of 1000 replicates.

**Diagnostic primers**

In order to provide diagnostic primers, two strategies were utilized. First published literature was searched for extracting the experimentally used primer pairs for the diagnosis of the Nipah virus along with relevant information. Secondly, putative primer pairs were also designed using the PrimerDesign-M tool (51), keeping default parameters primarily. Briefly, in the region of interest option, start and end of each genomic region were provided to design primers specific to the target gene. Further, we have chosen multiple fragments primer design options with flex parameter for fragment overlap option for each given genomic region. We have selected the primer length of 20 (minimum) to 25 (maximum) for each gene with the 5% detection limit. Then, complexity limit two was set to allow one degenerate position. Further, the maximum difference between melting temperatures (Tm) of reverse and forward primers was taken as 5°C. The window size of 10 was utilized for the investigation of dimerization, while the default dimer ratio (0.9) was chosen. Lastly, the G/C clamp option that helps to specify G or C at 3` ends of primer was selected. This helps to promote specific binding at 3` ends due to strong GC bases bonding.

**Vaccine Epitopes**

For the potential epitope identification, 9-mer overlapping peptides were generated for each NiV encoded proteins, i.e., N, P, W, V, C, M F, G, and L. Further the analyses were performed in quest to find promiscuous immune response, inducing peptides against the virus as also described previously (52). In order to have a proper immune response different arms of the immune system, i.e., B-cell epitopes, T-cell epitopes, and MHC binding is essential, hence considered in the study. The reliable and efficient linear B-cell epitopes of each NiV proteins were predicted using the Lbtope algorithm (53), and strict criteria of 60% were selected. The result is further analyzed and integrated on the webserver. Further, in order to identify the efficient MHC class-I binding peptides (putative cytotoxic T lymphocytes (CTL) epitopes) from the Nipah proteins, the Propred1 prediction server (54) was utilized, and preeminent 4% were selected. Similarly, MHC class-II binders (potential T helper (Th) epitopes) were estimated using the ProPred tool (55), and the uppermost three percent peptides were recommended as promiscuous binders. Furthermore, potential CTL epitopes were also derived using the CTLPred tool (56) developed using the artificial neural network (ANN) and support vector machine (SVM) techniques through employing the combined approach with the default cut-off of 0.51 for ANN and 0.36 for SVM. The top 3 epitopes were selected for each protein. Moreover, experimentally proven NiV epitopes were also searched.

**Small interfering RNAs (siRNAs) and MicroRNAs (miRNAs)**

RNA based therapeutics could provide an alternative way to fight against the pathogens. For the designing of siRNAs against the different NiV genes, two different algorithms, i.e., a virus-specific algorithm, VIRsiRNApred(57) and DesiRm(58) were used. Further, the immunomodulatory potential of the siRNAs is deduced using the imrna program (59). For the prediction of siRNAs using VIRsiRNApred, Model-2 was utilized. It is developed on 1725 viral siRNAs employing hybrid nucleotide frequencies, binary, and thermodynamic features. Further, only efficient siRNAs having at least 55% predicted inhibition score were considered. Additionally, off-targets were also elucidated against the Homo sapiens (human) genome assembly GRCh37 (hg19). Correspondingly, potential siRNAs using the threshold of 0.80 were also deduced applying the DesiRm algorithm. Moreover, the imRNA program with the “siRNA immunotoxicity” option along with the “screen siRNA library” module is used to explore the immunomodulatory or non-immunomodulatory potential of siRNAs. SiRNAs with a score of 4.5 and above are considered as immunomodulatory, and less than the threshold is non-immunomodulatory. Moreover, we have also predicted NiV miRNAs. First, the Vmir algorithm (60), which is consists of two programs VMir analyzer and VMir viewer, is used with the default settings to detect the putative precursor miRNAs (pre-miRNAs) hairpin (HP) structure. In brief, the maximum HP size of 200, minimum HP score (100), and minimum window count size of 35 are utilized. Further, these pre-miRNAs were subjected to the MatureBayes tool (61) to identify mature miRNAs.

**Single guide RNAs (sgRNAs) and genome editing**

Recently, clustered regularly interspaced short palindromic repeat-associated protein (CRISPR/Cas) system based genome editing employing sgRNAs also shown to have an application to target a particular genomic region (62) or viral pathogen (63). For this, we have used the ge-CRISPR tool (64) to screen the NiV genes/genome on both forward and reverse strands to discover and extract the possible sgRNAs, i.e., 20 base pair upstream sequences as putative targets based on the protospacer adjacent motif (PAM) mainly “NGG”.

**Web resource development**

The eventual goal is to provide the web resource of the putative therapeutic regimens and solutions from the study to assist in fighting with the deadly Nipah virus and support scientific society in therapeutic development. This platform, “NipahVR” is hosted on the Linux environment using LAMP (Linux, Apache HTTP Server, MySQL, and PHP) open-source web development platform. The front-end of the web interface is built using the PHP, HTML, CSS, and JavaScript as also accomplished earlier (52), and the back-end of the resource is complemented with MySQL for the data management.

**Results and Discussion**

NipahVR is an integrative and systematic resource mainly dedicated towards the putative therapeutics and vaccinome against the Nipah virus. A well-structured and dynamic web-interface is developed for navigation. It is classified into different divisions like genomes, phylogenomics, molecular diagnostic primers and most importantly vaccine epitopes (B-cell epitopes, CTL epitopes, MHC-I and -II binders), therapeutics (siRNAs, sgRNAs, miRNAs). The complete architecture of the NipahVR compendium is shown in **Figure 1,** demonstrating all the components.

**NipahVR genomes**

Genomic information of available 18 Nipah virus sequences was compiled and provided on the resource (**Table 1**). It is equipped with an advance genome search facility for easy navigation. Nipah genomes can be searched using different search options such as host/source (i.e., human, pig, bat), geographical region (Asia), and country (India, Bangladesh, Malaysia) with the detailed meta-information.

**Codon usage and context**

We have calculated the codon frequency and pattern in the genomes, which vary due to the nucleotide composition, GC percentage, expression level, etc. Additionally, codon preference is represented through histogram, where rare codons are shown in blue and black color signify preferred codons (**Figure 2**). The most preferred codons are AAA, AUG, UAA, and GCG, CGC, CGU is the least preferred or rare codons in the Nipah virus reference genome. Additionally, using the Anaconda software, we have also calculated codon pair residual values in the genome, indicating an association between two codons. Two-colored matrix depicting average residual values, red color denotes rare, and green color shows preferred codon pairs (**Figure 3**).

**Phylogenomics**

The reconstructed phylogenetic tree showed the sum of branch length of 0.11. Out of 15, 07 NiV genomes from Malaysia outbreaks (1998-99) were clustered together with the bootstrap of 100. Further, from the 04 NiV genomes from Bangladesh outbreak, two genomes from 2004 and 2010 outbreaks were clustered together. Interestingly, the 02 NiV genomes from the 2008 epidemic of Bangladesh were grouped with the NiV of 2007, West Bengal, India. Moreover, the recent outbreak of the NiV virus in Kerala, India, was found in the Indian and Bangladesh NiV genomes but at a distant branch. The phylogenetic reconstruction of NiV genomes is shown in **Figure 4**. The NiV genomes from Asian outbreaks are grouped according to their geographical location. The Malaysian outbreak of NiV(23) was closely related while the Indian and Bangladesh epidemic NiV displayed close resemblance with each other, except the recent Kerala outbreak, which is unrelated with all the NiV genomes. Our phylogenomic study showed that due to the course of time, the NiV showed significant mutations at the genomic level.

**Molecular diagnostics primers**

We have collected primer pairs utilized for the detection of the NiV. Detailed information about primers like the respective primer name, a sequence of primer, its orientation (forward and reverse), genomic region, and study reference is provided (**Table S1**). Overall, 55 forward and 53 reverse primers were reported against different genes of NiV and compiled on the webserver. Additionally, we have also designed primer pairs for each gene utilizing the PrimerDesign-M tool. In total, 2 primer pairs for N-gene; 6 primer pairs for P-gene; 3 primer pairs for M-gene; 6 primer pairs for F-gene; 1 primer pair for G-gene and 9 primer pairs for polymerase gene. Gene name, start-end, and melting temperature (Tm) of primers were reported. A detailed list of all predicted primer pairs is provided in **Table S2**. These primers (experimental and designed) will be valuable for the detection and diagnosis of NiV.

**Putative** epitome epitopes

In this study, efforts were made for the identification of potential vaccine candidates for the Nipah virus. Epitome Epitopes encompassing promising MHC I and II binders, CTL epitopes, and B-cell epitopes is cataloged. 9-mer peptides were generated from the Nipah proteins. Overall, 979 MHC-I and 1628 MHC-II binding peptides were deduced and presented on the server. For both MHC-I and II binders, peptide sequence, respective protein region, MHC-alleles, and counts were provided. The protein-wise number of peptides for both the MHC classes is shown in **Figure 5**. Likewise, 27 potential CTL epitopes with sequence, start-end, and alleles information were recorded (**Table 2**). Furthermore, 400 efficient B-cell epitopes (**Figure 6**) along with sequence, a b-cell confidence score is provided belonging to different Nipah proteins. Among all, for N protein EKKNNQDLK, P (W, V) protein SPEDEEPSS, for C protein LLTLFRRTK, for M protein AAYPLGVGK, for F protein SRLEDRRVR, for G protein DPLLAMDEG, and for L protein KLSQNLLVT peptides are the highest scoring and confident epitopes, which can be focused. Further, we have also found four experimentally proven linear b-cell epitopes, i.e., three for N protein (SIQTKFAP, SNRTQGE, and SPSAAE) and G (NQILKPKLISYTLPVVG). However, we did not find any T-cell epitopes.

Additionally, we have also analyzed putative epitopes from all the arms of the immune system, i.e., B-cell epitopes, MHC-I and II binders, and CTL epitopes to find the common epitopes, which could be promising and can be referred and recommended as the potential vaccine candidates (**Figure 7**). We have found the 24 epitopes reported to be B-cell epitope and also efficient MHC-I and II binders (**Table S3**). Likewise, 70 epitopes belonging to both b-cell as well as MHC-I binders (**Table S4**). Also, 109 epitopes are b-cell as well as MHC-II binders (**Table S5**). Further, there are two epitopes (“ILSAFNTVI” (G protein) and “FRRNNAIAF” (M protein)) which characterized in all three categories, i.e., CTL epitopes and both MHC-I and II binders. Likewise, 13 epitopes from CTL epitopes, and MHC-I binders, 5 epitopes from CTL epitopes and MHC-II binders were catered (**Table S6**). Furthermore, two epitopes were found to be as B-cell as well as CTL epitope, i.e., “QPSDDKRLS” from L protein and “NLRSRLAAK” from N protein. Along with this, 278 peptides that are putative MHC-I and II binders were also cataloged (**Table S7**).

**Small interfering RNAs and microRNAs**

RNA based therapeutic interventions provide another approach to counter lethal viruses through silencing the genes. In this study, we have also tried to provide a compendium of potent siRNAs against the individual NiV genes. Overall, 118 putative siRNAs with very few off-targets using the VIRsiRNApred algorithm, which is developed using the experimentally proven viral siRNAs and 441 siRNAs employing DesiRm tool were cataloged along with inferred inhibition efficiency in percentage. Additionally, the immunomodulatory potential of these siRNAs is also predicted, which could be helpful and crucial in the development of vaccine adjuvant or RNA based immunotherapy and therapeutics. Furthermore, the number of siRNA off-targets to the human genome is also presented. The resource provides a complete picture of these efficient siRNAs with a small number of off-targets and detail information such as sense-antisense sequence, gene region, start-end, efficacy scores, immunomodulatory potential, etc. The set of efficacious siRNAs, i.e., 18 using the VIRsiRNApred algorithm (**Table S8)** and 43 using DesiRm (**Table S9**) is specified.

Furthermore, we have also designed and identified the Nipah precursor and mature miRNAs. Totally, 22 precursor miRNAs (pre-miRNAs) were identified along with 44 mature Nipah-miRNAs (22 5p and 22 3p). Among these, 3 pre-miRNAs are from N, 3 from P/V/C, 1 from M, 2 from F, 3 from G, and 10 belong to the L gene. Detailed information related to miRNAs, i.e., mature miRNA sequences (5p and 3p), genomic region, the precursor (hairpin), and mature miRNA location on Nipah genome, precursor (hairpin) length, GC content, score, and rank is extracted and provided.

**Single guide RNAs (sgRNAs) and genome editing**

On the basis of our analysis, overall, 1412 sgRNAs from the NiV were obtained. Out of these, we have found and listed 126 sgRNAs that can act as putative targets against the virus. Apart from this, a list of the 21 most efficient sgRNAs is given in **Table 3**. The output of this displays sequence of sgRNA, associated PAM (NGG), information of strand (sense/antisense), start and end coordinates, AT and GC content, and efficiency of each sgRNA (percentage efficiency). This information will be beneficial to predict or identify CRISPR sgRNA targets against NiV and will certainly reduce experimental time and cost.

**Conclusion**

Nipah virus is a priority pathogen from the *Paramyxoviridae* family and a BSL-4 agent, which causes various diseases such as encephalitis, respiratory illness, and fever. Moreover, a high case fatality rate during epidemic, broad host range, and lack of therapeutics or prophylactic vaccines critically demand efforts and a multidisciplinary approach to develop combat strategies against this virus. Up to now, very few computational studies focusing on the NiV are performed, and there is no such kind of therapeutic web resource available for it. In the current work, we have developed all-inclusive resource “NipahVR” for the putative therapeutic solutions targeting individual Nipah genes and proteins. It provides a compendium of various components that includes genomics, diagnostic primers, vaccine epitome epitopes (MHC-I and -II binders, CTL epitopes, B-cell epitopes), and therapeutics (siRNAs, miRNAs, sgRNAs). Here, based on our analysis, we are also endorsing and providing a catalog of potential vaccine epitopes and efficient siRNAs, miRNAs, and sgRNAs. However, medicinal plant-based antivirals and other chemical compounds are not in the scope of current work. We anticipate that NipahVR will be useful and assist the wider scientific community in determining efficient antiviral candidates to fight against the Nipah and exterminate the infectivity. We will periodically update and maintain the stable functioning of the NipahVR web resource.

**Acknowledgements**

We would like to acknowledge Department of Biotechnology (DBT), Government of India [GAP0001] for infrastructure support.

**Funding**

This work was supported by the Council of Scientific and Industrial Research (CSIR), Government of India [OLP0501, OLP0143].

**Author Contributions**

This study is conceived, designed and supervised by MK. genomic data collection and curation; AKG, web server development; AKG, codon analysis; AKG, vaccine epitope analysis; AK, KM, AKG, phylogenetic analysis; AR, sgRNAs and diagnostic primers; KK, siRNA analysis; SD, miRNA analysis; AT, data interpretation; AKG, AK, AR, MK, Manuscript writing, AKG, AR, KK, MK.

**Competing interests**

The authors declare that they have no competing interests.

**References:**

1. Harcourt, B.H., Tamin, A., Ksiazek, T.G.*, et al.* (2000) Molecular characterization of Nipah virus, a newly emergent paramyxovirus. *Virology*, **271**, 334-349.

2. Wang, L., Harcourt, B.H., Yu, M.*, et al.* (2001) Molecular biology of Hendra and Nipah viruses. *Microbes Infect*, **3**, 279-287.

3. Chua, K.B., Bellini, W.J., Rota, P.A.*, et al.* (2000) Nipah virus: a recently emergent deadly paramyxovirus. *Science*, **288**, 1432-1435.

4. Bonaparte, M.I., Dimitrov, A.S., Bossart, K.N.*, et al.* (2005) Ephrin-B2 ligand is a functional receptor for Hendra virus and Nipah virus. *Proc Natl Acad Sci U S A*, **102**, 10652-10657.

5. Negrete, O.A., Levroney, E.L., Aguilar, H.C.*, et al.* (2005) EphrinB2 is the entry receptor for Nipah virus, an emergent deadly paramyxovirus. *Nature*, **436**, 401-405.

6. Tamin, A., Harcourt, B.H., Ksiazek, T.G.*, et al.* (2002) Functional properties of the fusion and attachment glycoproteins of Nipah virus. *Virology*, **296**, 190-200.

7. Sharma, V., Kaushik, S., Kumar, R.*, et al.* (2019) Emerging trends of Nipah virus: A review. *Rev Med Virol*, **29**, e2010.

8. Lo Presti, A., Cella, E., Giovanetti, M.*, et al.* (2016) Origin and evolution of Nipah virus. *J Med Virol*, **88**, 380-388.

9. Chua, K.B., Goh, K.J., Wong, K.T.*, et al.* (1999) Fatal encephalitis due to Nipah virus among pig-farmers in Malaysia. *Lancet*, **354**, 1257-1259.

10. Ang, B.S.P., Lim, T.C.C., Wang, L. (2018) Nipah Virus Infection. *J Clin Microbiol*, **56**.

11. Luby, S.P., Rahman, M., Hossain, M.J.*, et al.* (2006) Foodborne transmission of Nipah virus, Bangladesh. *Emerg Infect Dis*, **12**, 1888-1894.

12. Luby, S.P., Gurley, E.S., Hossain, M.J. (2009) Transmission of human infection with Nipah virus. *Clin Infect Dis*, **49**, 1743-1748.

13. Drexler, J.F., Corman, V.M., Muller, M.A.*, et al.* (2012) Bats host major mammalian paramyxoviruses. *Nat Commun*, **3**, 796.

14. Epstein, J.H., Prakash, V., Smith, C.S.*, et al.* (2008) Henipavirus infection in fruit bats (Pteropus giganteus), India. *Emerg Infect Dis*, **14**, 1309-1311.

15. Yadav, P.D., Raut, C.G., Shete, A.M.*, et al.* (2012) Detection of Nipah virus RNA in fruit bat (Pteropus giganteus) from India. *Am J Trop Med Hyg*, **87**, 576-578.

16. Reynes, J.M., Counor, D., Ong, S.*, et al.* (2005) Nipah virus in Lyle's flying foxes, Cambodia. *Emerg Infect Dis*, **11**, 1042-1047.

17. Wacharapluesadee, S., Lumlertdacha, B., Boongird, K.*, et al.* (2005) Bat Nipah virus, Thailand. *Emerg Infect Dis*, **11**, 1949-1951.

18. Chadha, M.S., Comer, J.A., Lowe, L.*, et al.* (2006) Nipah virus-associated encephalitis outbreak, Siliguri, India. *Emerg Infect Dis*, **12**, 235-240.

19. Ksiazek, T.G., Rota, P.A., Rollin, P.E. (2011) A review of Nipah and Hendra viruses with an historical aside. *Virus Res*, **162**, 173-183.

20. Peterson, A.T. (2015) Mapping risk of Nipah virus transmission across Asia and across Bangladesh. *Asia Pac J Public Health*, **27**, NP824-832.

21. Ambat, A.S., Zubair, S.M., Prasad, N.*, et al.* (2019) Nipah virus: A review on epidemiological characteristics and outbreaks to inform public health decision making. *J Infect Public Health*, **12**, 634-639.

22. Harit, A.K., Ichhpujani, R.L., Gupta, S.*, et al.* (2006) Nipah/Hendra virus outbreak in Siliguri, West Bengal, India in 2001. *Indian J Med Res*, **123**, 553-560.

23. Arankalle, V.A., Bandyopadhyay, B.T., Ramdasi, A.Y.*, et al.* (2011) Genomic characterization of Nipah virus, West Bengal, India. *Emerg Infect Dis*, **17**, 907-909.

24. Arunkumar, G., Chandni, R., Mourya, D.T.*, et al.* (2019) Outbreak Investigation of Nipah Virus Disease in Kerala, India, 2018. *J Infect Dis*, **219**, 1867-1878.

25. Yadav, P.D., Shete, A.M., Kumar, G.A.*, et al.* (2019) Nipah Virus Sequences from Humans and Bats during Nipah Outbreak, Kerala, India, 2018. *Emerg Infect Dis*, **25**, 1003-1006.

26. Gurley, E.S., Montgomery, J.M., Hossain, M.J.*, et al.* (2007) Person-to-person transmission of Nipah virus in a Bangladeshi community. *Emerg Infect Dis*, **13**, 1031-1037.

27. Ravichandran, L., Venkatesan, A., Febin Prabhu Dass, J. (2018) Epitope-based immunoinformatics approach on RNA-dependent RNA polymerase (RdRp) protein complex of Nipah virus (NiV). *J Cell Biochem*.

28. Satterfield, B.A., Dawes, B.E., Milligan, G.N. (2016) Status of vaccine research and development of vaccines for Nipah virus. *Vaccine*, **34**, 2971-2975.

29. Mungall, B.A., Middleton, D., Crameri, G.*, et al.* (2006) Feline model of acute nipah virus infection and protection with a soluble glycoprotein-based subunit vaccine. *J Virol*, **80**, 12293-12302.

30. DeBuysscher, B.L., Scott, D., Marzi, A.*, et al.* (2014) Single-dose live-attenuated Nipah virus vaccines confer complete protection by eliciting antibodies directed against surface glycoproteins. *Vaccine*, **32**, 2637-2644.

31. Broder, C.C., Weir, D.L., Reid, P.A. (2016) Hendra virus and Nipah virus animal vaccines. *Vaccine*, **34**, 3525-3534.

32. Walpita, P., Cong, Y., Jahrling, P.B.*, et al.* (2017) A VLP-based vaccine provides complete protection against Nipah virus challenge following multiple-dose or single-dose vaccination schedules in a hamster model. *NPJ Vaccines*, **2**, 21.

33. Broder, C.C., Xu, K., Nikolov, D.B.*, et al.* (2013) A treatment for and vaccine against the deadly Hendra and Nipah viruses. *Antiviral Res*, **100**, 8-13.

34. Geisbert, T.W., Mire, C.E., Geisbert, J.B.*, et al.* (2014) Therapeutic treatment of Nipah virus infection in nonhuman primates with a neutralizing human monoclonal antibody. *Sci Transl Med*, **6**, 242ra282.

35. Mungall, B.A., Schopman, N.C., Lambeth, L.S.*, et al.* (2008) Inhibition of Henipavirus infection by RNA interference. *Antiviral Res*, **80**, 324-331.

36. Chong, H.T., Kamarulzaman, A., Tan, C.T.*, et al.* (2001) Treatment of acute Nipah encephalitis with ribavirin. *Ann Neurol*, **49**, 810-813.

37. Georges-Courbot, M.C., Contamin, H., Faure, C.*, et al.* (2006) Poly(I)-poly(C12U) but not ribavirin prevents death in a hamster model of Nipah virus infection. *Antimicrob Agents Chemother*, **50**, 1768-1772.

38. Dawes, B.E., Kalveram, B., Ikegami, T.*, et al.* (2018) Favipiravir (T-705) protects against Nipah virus infection in the hamster model. *Sci Rep*, **8**, 7604.

39. Lin, L.T., Hsu, W.C., Lin, C.C. (2014) Antiviral natural products and herbal medicines. *J Tradit Complement Med*, **4**, 24-35.

40. Dhama, K., Karthik, K., Khandia, R.*, et al.* (2018) Medicinal and Therapeutic Potential of Herbs and Plant Metabolites / Extracts Countering Viral Pathogens - Current Knowledge and Future Prospects. *Curr Drug Metab*, **19**, 236-263.

41. Niedermeier, S., Singethan, K., Rohrer, S.G.*, et al.* (2009) A small-molecule inhibitor of Nipah virus envelope protein-mediated membrane fusion. *J Med Chem*, **52**, 4257-4265.

42. Hotard, A.L., He, B., Nichol, S.T.*, et al.* (2017) 4'-Azidocytidine (R1479) inhibits henipaviruses and other paramyxoviruses with high potency. *Antiviral Res*, **144**, 147-152.

43. Sakib, M.S., Islam, M.R., Hasan, A.K.*, et al.* (2014) Prediction of epitope-based peptides for the utility of vaccine development from fusion and glycoprotein of nipah virus using in silico approach. *Adv Bioinformatics*, **2014**, 402492.

44. Saha, C.K., Mahbub Hasan, M., Saddam Hossain, M.*, et al.* (2017) In silico identification and characterization of common epitope-based peptide vaccine for Nipah and Hendra viruses. *Asian Pac J Trop Med*, **10**, 529-538.

45. Rajput, A., Kumar, A., Kumar, M. (2019) Computational Identification of Inhibitors Using QSAR Approach Against Nipah Virus. *Frontiers in Pharmacology*, **10**.

46. Luby, S.P. (2013) The pandemic potential of Nipah virus. *Antiviral Res*, **100**, 38-43.

47. Moura, G., Pinheiro, M., Silva, R.*, et al.* (2005) Comparative context analysis of codon pairs on an ORFeome scale. *Genome Biol*, **6**, R28.

48. Pickett, B.E., Sadat, E.L., Zhang, Y.*, et al.* (2012) ViPR: an open bioinformatics database and analysis resource for virology research. *Nucleic Acids Res*, **40**, D593-598.

49. Masson, P., Hulo, C., De Castro, E.*, et al.* (2013) ViralZone: recent updates to the virus knowledge resource. *Nucleic Acids Res*, **41**, D579-583.

50. Kumar, S., Stecher, G., Tamura, K. (2016) MEGA7: Molecular Evolutionary Genetics Analysis Version 7.0 for Bigger Datasets. *Mol Biol Evol*, **33**, 1870-1874.

51. Yoon, H., Leitner, T. (2015) PrimerDesign-M: a multiple-alignment based multiple-primer design tool for walking across variable genomes. *Bioinformatics*, **31**, 1472-1474.

52. Gupta, A.K., Kaur, K., Rajput, A.*, et al.* (2016) ZikaVR: An Integrated Zika Virus Resource for Genomics, Proteomics, Phylogenetic and Therapeutic Analysis. *Sci Rep*, **6**, 32713.

53. Singh, H., Ansari, H.R., Raghava, G.P. (2013) Improved method for linear B-cell epitope prediction using antigen's primary sequence. *PLoS One*, **8**, e62216.

54. Singh, H., Raghava, G.P. (2003) ProPred1: prediction of promiscuous MHC Class-I binding sites. *Bioinformatics*, **19**, 1009-1014.

55. Singh, H., Raghava, G.P. (2001) ProPred: prediction of HLA-DR binding sites. *Bioinformatics*, **17**, 1236-1237.

56. Bhasin, M., Raghava, G.P. (2004) Prediction of CTL epitopes using QM, SVM and ANN techniques. *Vaccine*, **22**, 3195-3204.

57. Qureshi, A., Thakur, N., Kumar, M. (2013) VIRsiRNApred: a web server for predicting inhibition efficacy of siRNAs targeting human viruses. *J Transl Med*, **11**, 305.

58. Ahmed, F., Raghava, G.P. (2011) Designing of highly effective complementary and mismatch siRNAs for silencing a gene. *PLoS One*, **6**, e23443.

59. Chaudhary, K., Nagpal, G., Dhanda, S.K.*, et al.* (2016) Prediction of Immunomodulatory potential of an RNA sequence for designing non-toxic siRNAs and RNA-based vaccine adjuvants. *Sci Rep*, **6**, 20678.

60. Sullivan, C.S., Grundhoff, A. (2007) Identification of viral microRNAs. *Methods Enzymol*, **427**, 3-23.

61. Gkirtzou, K., Tsamardinos, I., Tsakalides, P.*, et al.* (2010) MatureBayes: a probabilistic algorithm for identifying the mature miRNA within novel precursors. *PLoS One*, **5**, e11843.

62. Sander, J.D., Joung, J.K. (2014) CRISPR-Cas systems for editing, regulating and targeting genomes. *Nat Biotechnol*, **32**, 347-355.

63. Price, A.A., Sampson, T.R., Ratner, H.K.*, et al.* (2015) Cas9-mediated targeting of viral RNA in eukaryotic cells. *Proc Natl Acad Sci U S A*, **112**, 6164-6169.

64. Kaur, K., Gupta, A.K., Rajput, A.*, et al.* (2016) ge-CRISPR - An integrated pipeline for the prediction and analysis of sgRNAs genome editing efficiency for CRISPR/Cas system. *Sci Rep*, **6**, 30870.

**Figures and Tables**

**Figure 1.** Overview of NipahVR resource components

**Figure 2**. Histogram showing codon distribution (rare (blue) and preferred (black)) of NiV genome

**Figure 3**. Matrix illustrating codon context analysis

**Figure 4**. Phylogenetic tree showing the relationship of 15 NiV complete genomes employing Neighbor-joining method

**Figure 5**. Chart displaying the number of MHC-I and II binders from different proteins

**Figure 6**. Pie chart-showing number of putative b-cell epitopes for individual Nipah proteins

**Figure 7**. Venn diagram showing the number of peptides/epitopes belonging and common in diverse epitope classes

**Table 1**. List of 18 Nipah virus genomes

**Table 2**. Table showing potential 27 CTL epitopes against individual NiV proteins along with detail information

**Table 3**. List of 21 efficient sgRNAs targeting Nipah virus genes

**Supplementary Information**

**Table S1.** List of experimentally used primers for the detection of Nipah virus

**Table S2.** Catalog of designed primer pairs for the detection of Nipah virus

**Table S3**. 24 efficient B-cell epitopes and MHC-I /II binders

**Table S4.**70 B-cell epitopes as well as efficient MHC-I binders

**Table S5.** 109 B-cell epitopes as well as efficient MHC-II binders

**Table S6.** List of 16 CTL epitopes as well as MHC-I/II binders

**Table S7.** Catalog of 278 efficient MHC-I and II binders

**Table S8**. List of potential siRNAs utilizing the VIRsiRNApred algorithm

**Table S9**. List of potential siRNAs utilizing the DesiRm algorithm
