# Supplementary material for: NipahVR: a resource of multi-targeted putative therapeutics and epitopes for the Nipah virus
Source: Database (Oxford). 2020 Feb 24;2020:baz159. doi: 10.1093/database/baz159 (PMC7036594; doi:10.1093/database/baz159)
Supplement: Supplementary_Information_baz159 [file supplementary_information_baz159.docx]

**Supplementary Information**

**NipahVR: a resource of multi-targeted putative therapeutics and epitopes for the nipah virus**

Amit Kumar Gupta, Archit Kumar, Akanksha Rajput, Karambir Kaur, Showkat Ahmed Dar, Anamika Thakur, Kirti Megha and Manoj Kumar*

Virology Unit and Bioinformatics Centre, Institute of Microbial Technology, Council of Scientific and Industrial Research (CSIR), Sector 39-A, Chandigarh-160036, India

* To whom correspondence should be addressed. Tel, 91-172-6665453; Fax, 91-172-2690585; 91-172-2690632; Email, [manojk@imtech.res.in](mailto:manojk@imtech.res.in)

**Table S1.** List of experimentally used primers for the detection of Nipah virus

| **Gene** | **F Primer name** | **F Sequence** | **R Primer name** | **R Sequence** | **Technique** | **PMID** |
| --- | --- | --- | --- | --- | --- | --- |
| N | Ni-NP1209 | GCAAGAGAGTAATGTTCAGGCTAGAG | Ni- NP1314 | CTGTTCTATAGGTTCTTCCCCTTCAT | RT-PCR | 15288966 |
| N | F1 | GGCAGGATTCTTCGCAACCATC | R1 | GGCTCTTGG GCCAATTTCTCTG | RT-qPCR | 21593145 |
| M | F2 | AACGGCTGTTTGCTCAAATGGG | R2 | GCTGCTACTCGGCTGATCTCAC | RT-qPCR | 21593145 |
| F | F3 | GCAGGGC AATCTCACAATCAGG | R3 | GGACCGATAGCAATGCCTTCAG | RT-qPCR | 21593145 |
| G | F4 | AGGTTCAAAGATCAGCCAGTCG | R4 | AAAGGGAGTGGGTTAGGACAAG | RT-qPCR | 21593145 |
| L | F5 | ATGGTGCTGTGCTGTCTCAGG | R5 | AGCCGACATTTCTTGACAACCC | RT-qPCR | 21593145 |
| N | NiV01 | TAGAAATAATCTCAGACATCGGAAA | NiV02 | CCCATAGACCTGTCAATAGTAGTAGC | RT-PCR | 16912918 |
| N | NIP-NF3 | GGCTAGAGAGGCAAAATTTGCTGC | NIP-NR1 | ACCGGATGTGCTCACAGAACTG | RT-PCR | 16784519 |
| N | F1 | ACCAAACAAGGGAAAATATGGAT | R1 | CTCGGCTGCACTCGGAGATCTGAT | RT-PCR | 21529409 |
| N | F2 | TATGGATACGTTAAAATATAT | R2 | TGATAACTGCAGAGACCAGAGT | RT-PCR | 21529409 |
| N | F3 | TCAAGACTGCTCGGGACAGCAG | R3 | TCAATAGTAGTAGCCACACCCAT | RT-PCR | 21529409 |
| N | F4 | GCTTGATGCTACTCTACAGAG | R4 | TGATTGCTGGGTCATCTGTTGCATT | RT-PCR | 21529409 |
| N | F5 | TGACAGTGTGCCGAGCAGTTCTGT | R5 | TAAGAGGTATTGTATACTCCAGT | RT-PCR | 21529409 |
| N | F6 | CACACTACACTCTAATAAAGAT | - | - | RT-PCR | 21529409 |
| P | F7 | TGCAGTGCACCAGTGGAGAATCT | R6 | CACCTCCATCATCCTTAGAC | RT-PCR | 21529409 |
| P | F8 | TTCTCCTGTGATTGCTGAACACT | R7 | TCCAAATTGACATTTCTGTTAGT | RT-PCR | 21529409 |
| P | F9 | GATAAGACTGAGATCACCAGCGAT | R8 | CAGATGGATATTTCTCGTCTGT | RT-PCR | 21529409 |
| P | F10 | CTTGAGTCTATTGACAGGGTTCT | R9 | TCACTGGTTTAAGCTCAGGATT | RT-PCR | 21529409 |
| P | F11 | TCTTCTACATGTAGACAGTAG | R10 | TCATGCTACATACACTTCAAT | RT-PCR | 21529409 |
| M | F12 | GAGCATTTCAAGTGAGTCT | R11 | TTATCAAGATACCCACCATTCT | RT-PCR | 21529409 |
| M | F13 | TTGATCAGATACAGCTCGAC | R12 | TAGTTCGTGGAATCATGTAGATT | RT-PCR | 21529409 |
| M | F14 | GCCTTCTGTTCCGAGAGAGTT | R13 | TGTATTGTCAATGAAGACATCAT | RT-PCR | 21529409 |
| F | F15 | GTTGGTAGACCTATCAATCATAT | R14 | CACTGCACTCCGAGATCATC | RT-PCR | 21529409 |
| F | F16 | GCAGCATAGAATCAACTAATG | R15 | GCGGTCAGTACATAGACTGT | RT-PCR | 21529409 |
| F | F17 | TCCAACAGGCCTATATCCAAG | R16 | TGCTGATCCATTCTGAATTGT | RT-PCR | 21529409 |
| F | F18 | GCTCAACGACTCCTTGATACTGTT | R17 | TACAGTATGATCATAGACAACAT | RT-PCR | 21529409 |
| F | F19 | TGTACTTGCAATTATACATTGT | R18 | GATCCATTGTAATCATAGTG | RT-PCR | 21529409 |
| G | F20 | TATCCAATGAGTTATGGACCT | R19 | TACTTTGCATATAGCCACCTG | RT-PCR | 21529409 |
| G | F21 | TGCTGGATACTCTGCAATGCAT | R20 | TCGCAGAGTGTCAATACAGCAAACCT | RT-PCR | 21529409 |
| G | F22 | TGTCTAGTACCTCTCCAACTCCT | R21 | GCAACCAGACCGCAGAGAATCCT | RT-PCR | 21529409 |
| G | F23 | TATAATGACTGTTTGGTCTAAT | R22 | TCAGAGTTAACAGTCTATACAT | RT-PCR | 21529409 |
| G | F24 | TACTTCATTATCTTTGAATACAG | R23 | CATTGATTGTCATCACTATGC | RT-PCR | 21529409 |
| P | F25 | TGCATATTGCGTACCCTGAATGT | R24 | TGTTATCAAGTTTGCTAGTCAT | RT-PCR | 21529409 |
| P | F26 | GGATGATGATGGAGACAACAAT | R25 | GAGGGCATTGGACCTCGAGATT | RT-PCR | 21529409 |
| P | F27 | GCACATGCATCTAAGCATAT | R26 | TTCCCAGTTCTTGACACAATCATC | RT-PCR | 21529409 |
| P | F28 | TCAGTTCCTCGTGGAAACAGTC | R27 | TATATTATTGATGGATTGAGGAT | RT-PCR | 21529409 |
| P | F29 | GATGATATATTCATTCATTATCCT | R28 | TCTCATAGGCACTCAAGAAT | RT-PCR | 21529409 |
| P | F30 | TCAAGGAATGTCGGCTATTGTAT | R29 | TCAGTTGATATAAGGAGTTGCT | RT-PCR | 21529409 |
| P | F31 | TAGCTAGCTTCCTGATGGACAGG | R30 | TTGTCCAGTATCTCATGAGCGG | RT-PCR | 21529409 |
| P | F32 | GTACAGATGAGAGATCAGATAT | R31 | TACTGTCGCAATCCTGATAGCAG | RT-PCR | 21529409 |
| P | F33 | TGATCCAGATCCTGTTTCAG | R32 | TGAAGCTCCTCAGTTGACCAT | RT-PCR | 21529409 |
| P | F34 | CTGTGATTAACCTACGAGAGGATAT | R33 | TTCAGATCTATTATCCAAGGAGG | RT-PCR | 21529409 |
| P | F35 | CAGGTCAGAGAGAACTGAAGCT | R34 | TCAGCAATCGAGTATTCGGATGG | RT-PCR | 21529409 |
| P | F36 | TCTCAAGATTATTTAACATGT | R35 | TAGAATCTGGGTTGCTATACACT | RT-PCR | 21529409 |
| P | F37 | TTCACATCATTTGGAACCGTAT | - | - | RT-PCR | 21529409 |
| N | NVBNF2B | CTGGTCTCTGCAGTTATCACCATCGA | NVBN593R | ACGTACTTAGCCCATCTTCTAGTTTCA | rRT-PCR | 22304936, 26491176, 29260663 |
| N | NVBN5NCF1 | GGTCTTGGTATTGGATCCTC | NVBN3NCR1 | GTTTAATCTAAGTTAAGATTG | rRT-PCR | 22304936 |
| P | NVBPPCRFW | AGCAGTTATCAGCTGGGAGTTCAACTTAC | NVBPPCRREV | ATGCGTGAATGAACTACAATACGAATCGAC | rRT-PCR | 22304936 |
| M | NVBMPCRFW | TCCAATAACTGGTCAATTGAGGACAGAAATCCTG | NVBMPCRREV | CATAATAGTTGTCTAATTATTAACCGAATATTCAC | rRT-PCR | 22304936 |
| F | NVBPCRFFW | CAAGCATTATTACTATCTGATCAACAAAAGGATTGG | NVBPCRFREV | GAATATCAACTGTTCATTCATGGTTGAGTAC | rRT-PCR | 22304936 |
| G | NVBPCRGFW | CAGGTCCATAACTCATTGGATATTAAACTGTGTCC | NVBPCRGREV | CAAGATTTAGCTCTACTATATCAAATGGAGTTTCAGTCAAG | rRT-PCR | 22304936 |
| L | NVBPCRL1FW | CAGGTCCTTGATTGTGCTAATTTTCTTGAG | FRAG4REV | GATCTTATCAGGCCTTTAGTTGTATCTAATAGACC | rRT-PCR | 22304936 |
| L | FRAG5FW | TGAGGACCTTGAACTAGCTAGCTTCCT | NVBLREV | AATTGTCGGTCGGTTCTGGACTTGGAAGATCAAATCAGATAATGGATATG | rRT-PCR | 22304936 |
| N | N1198F | TCAGCAGGAAGGCAAGAGAGTAA | N1297R | CCCCTTCATCGATATCTTGATCA | Taqman PCR | 23894501 |
| L | F1 | CAAAACAGAGATGCGAGCAG | R1 | ATGCATGAATCTGAACGGAA | qRT-PCR | 25059478 |

**Table S2.** Catalog of designed primer pairs for the detection of Nipah virus

| **Gene** | **F Primer ID** | **F sequence** | **start** | **stop** | **Tm** | **R Primer ID** | **R sequence** | **start** | **stop** | **Tm** |
| --- | --- | --- | --- | --- | --- | --- | --- | --- | --- | --- |
| N | N.F1 | CTGCTCTCCCAGAGTCTATCAG | 770 | 791 | 61.15 | N.R1 | ACCAGTTTCCTCGACATAGTTTCC | 901 | 878 | 61.83 |
| N | N.F2 | CTATCAGTAAGGAAGTTCATGGTTG | 785 | 809 | 59.16 | N.R2 | GTGCTGATTTTTGGCCTAGTC | 1214 | 1194 | 59.38 |
| P | P.F1 | CAACTTGCAAAACTCTAACCTTC | 2362 | 2384 | 58.18 | P.R1 | TCGTCCGTATGTCTTCTGTATTTC | 2495 | 2472 | 59.79 |
| P | P.F2 | ATGATCATGGAGGAGAATGTACCG | 2755 | 2778 | 61.36 | P.R2 | GGCTCTTCATCTTCAGGAGAAATG | 3220 | 3197 | 61.26 |
| P | P.F3 | TCGGCATTTCTCCTGAAGATG | 3193 | 3213 | 59.78 | P.R3 | GTGGTTCTTCCTTCACTGCTG | 3366 | 3346 | 61.08 |
| P | P.F4 | TATCATTGGAGCATCGAGAGG | 3495 | 3515 | 58.43 | P.R4 | GGAAGAAAGTGTTTGAGAAATCATC | 3873 | 3849 | 58.48 |
| P | P.F5 | GACACTGATCGCTTGAATTATCAC | 3879 | 3902 | 59.8 | P.R5 | AATCATCTGCCATAGGAACAAATTG | 4380 | 4356 | 59.72 |
| P | P.F6 | GAGCAGCAATCTCTTTTTTC | 4215 | 4234 | 54.83 | P.R6 | TGAGGTTGTTATTATTGACCTG | 4621 | 4600 | 55.44 |
| M | M.F1 | CCCTCAGACGATAACACAATACTTG | 4657 | 4681 | 61.14 | M.R1 | GGGCTGAAATCAGATACTCCTTC | 5169 | 5147 | 60.56 |
| M | M.F2 | ATTGTTGTTTTTGTCATGATCATTC | 4845 | 4869 | 56.93 | M.R2 | ACTAGGGCTGAAATCAGATAC | 5173 | 5153 | 56.28 |
| M | M.F3 | TGAACCAGAAATTGATGAAAATGG | 5206 | 5229 | 57.62 | M.R3 | CCACTAGTCAGTACTTTCTTCC | 5550 | 5529 | 57.12 |
| F | F.F1 | AAGGAGTAACAAGAAAATAC | 6772 | 6791 | 49.74 | F.R1 | TGTTAAGATACCGTTTAATC | 6917 | 6898 | 49.59 |
| F | F.F2 | ATAAAAATGATTCCGAATGTGTC | 6828 | 6850 | 55.14 | F.R2 | TTGAATTAGAAACTGGGTCTTG | 7329 | 7308 | 55.9 |
| F | F.F3 | CTAATGAAGCTGTCGTTAAACTTC | 7114 | 7137 | 57.84 | F.R3 | ACCTGTTATGCTGTCACTTTC | 7445 | 7425 | 57.81 |
| F | F.F4 | GCAGCATTGAATCAACTAATGAAG | 7099 | 7122 | 58.75 | F.R4 | TGATTTGACCTGTTATGCTGTC | 7452 | 7431 | 58.27 |
| F | F.F5 | TTCATCACATGTTCCCAGATTTG | 7760 | 7782 | 58.96 | F.R5 | CTGATTCATGCTGGATATCTGAC | 8048 | 8026 | 58.65 |
| F | F.F6 | GGATCGACTGAGAAGTGTCC | 7725 | 7744 | 59.02 | F.R6 | GGTGGTGTTGTCAATCATCAG | 7901 | 7881 | 59.05 |
| G | G.F1 | CCTTTTAGAGAGTATAGGCCACAG | 9441 | 9464 | 60.03 | G.R1 | CAGCACTACAGTGGTAAACGG | 9795 | 9775 | 60.4 |
| L | L.F1 | GATATTAACTGAGACTCCACTTG | 11147 | 11169 | 55.99 | L.R1 | GTCTATGGTCAATTGAATGATTTC | 11317 | 11294 | 55.92 |
| L | L.F2 | ATTCCCTTAAAAACTGAATACC | 11379 | 11400 | 53.43 | L.R2 | GCTTTGAATGACTTTTTCATG | 11761 | 11741 | 53.84 |
| L | L.F3 | GGAATTCATGCATATTGCGTACC | 11654 | 11676 | 59.88 | L.R3 | ATCCGATTTGACTGTTGTCTCC | 12176 | 12155 | 59.68 |
| L | L.F4 | TCTTTCGTACGTTTGGCCATC | 12487 | 12507 | 60.44 | L.R4 | AATTGTTCATCCTCGAGATAAGCAC | 13015 | 12991 | 60.91 |
| L | L.F5 | ATACAACATGCTGGAATATGTC | 12962 | 12983 | 55.88 | L.R5 | TAAAATATTTACCGACACCTGAG | 13149 | 13127 | 55.69 |
| L | L.F6 | GCATGTCAAGTCATAGCAGAG | 13095 | 13115 | 58.56 | L.R6 | GTGATTGTGAGGATTAAACTCGG | 13481 | 13459 | 58.91 |
| L | L.F7 | CGAATCAATGGCTATATTTG | 13607 | 13626 | 51.67 | L.R7 | GAATTTTCTTCGAATAAATAAAAAG | 14097 | 14073 | 51.64 |
| L | L.F8 | TCACCGAACCCAATGCTAAAAG | 14661 | 14682 | 60.82 | L.R8 | CCAATTGGGAATCCCTAGGTAC | 15183 | 15162 | 60.09 |
| L | L.F9 | AGATTATAACAAAGGCTGACAAGG | 15925 | 15948 | 58.27 | L.R9 | TATCCTCTCGTAGACTTATCACAG | 16398 | 16375 | 58.15 |

**Table S3.** 24 efficient B-cell epitopes and MHC-I/II binders

| **Sr. No.** | **Protein** | **Epitopes (B-cell+MHC-I/II binders)** |
| --- | --- | --- |
| 1 | C | LTLFRRTKK |
| 2 | F | ILHYEKLSK |
| 3 | F | IYKNNTHDL |
| 4 | F | VKGVTRKYK |
| 5 | G | IMIIQNYTR |
| 6 | L | FFVPRDSQL |
| 7 | L | LILDFRSKL |
| 8 | L | LPRAAHEIL |
| 9 | L | METTVKSDI |
| 10 | L | VMMEDGLLV |
| 11 | M | IFFLSITKL |
| 12 | M | IRTIAAYPL |
| 13 | M | YPLGVGKSA |
| 14 | N | FAPGGYPLL |
| 15 | N | LKTARDSSK |
| 16 | N | YPLLWSFAM |
| 17 | P | LRNLSDPAK |
| 18 | P | VKEEPPQKR |
| 19 | V | LRNLSDPAK |
| 20 | V | VERRNLEDL |
| 21 | V | VKEEPPQKR |
| 22 | W | LRNLSDPAK |
| 23 | W | VERRNLEDL |
| 24 | W | VKEEPPQKR |

**Table S4.** 70 B-cell epitopes as well as efficient MHC-I binders

| **Sr. No.** | **Protein** | **Epitopes**  **(B-cell+MHC-I binder)** | **Sr. No.** | **Protein** | **Epitopes**  **(B-cell+MHC-I binder)** | **Sr. No.** | **Protein** | **Epitopes**  **(B-cell+MHC-I binder)** |
| --- | --- | --- | --- | --- | --- | --- | --- | --- |
| 1 | C | EENVPDMDL | 25 | L | METTVKSDI | 49 | P | VKEEPPQKR |
| 2 | C | LTLFRRTKK | 26 | L | SDIKYQPLI | 50 | V | AQPPYHWSI |
| 3 | F | AFGGNYETL | 27 | L | SQNLLVTSY | 51 | V | EIQKTYGRS |
| 4 | F | EKKRNTYSR | 28 | L | TEPRRLVDV | 52 | V | EPPQKRLPM |
| 5 | F | FGPNLQDPV | 29 | L | TIVPPQKVL | 53 | V | KEDRETDLV |
| 6 | F | GLVKGVTRK | 30 | L | VMMEDGLLV | 54 | V | KEEPPQKRL |
| 7 | F | ILHYEKLSK | 31 | M | HPQDLLEEL | 55 | V | LRNLSDPAK |
| 8 | F | IYKNNTHDL | 32 | M | IFFLSITKL | 56 | V | RRNLEDLSS |
| 9 | F | KKRNTYSRL | 33 | M | IRTIAAYPL | 57 | V | SRITPLPRR |
| 10 | F | NTYSRLEDR | 34 | M | KYYSVDYCR | 58 | V | VERRNLEDL |
| 11 | F | NYETLLRTL | 35 | M | VSDFSPSSW | 59 | V | VKEEPPQKR |
| 12 | F | VKGVTRKYK | 36 | M | YPLGVGKSA | 60 | W | AQPPYHWSI |
| 13 | G | CKFTLPPLK | 37 | N | FAPGGYPLL | 61 | W | DHPPTKKAR |
| 14 | G | IMIIQNYTR | 38 | N | KFAPGGYPL | 62 | W | EIQKTYGRS |
| 15 | G | KCKFTLPPL | 39 | N | LKTARDSSK | 63 | W | EPPQKRLPM |
| 16 | G | QYSKPENCR | 40 | N | NLRSRLAAK | 64 | W | GMFEDHPPT |
| 17 | G | RLSIGSPSK | 41 | N | SEKKNNQDL | 65 | W | HPPTKKARV |
| 18 | L | AYPECNNIL | 42 | N | YPLLWSFAM | 66 | W | KEDRETDLV |
| 19 | L | DSQLDQVDR | 43 | P | EIQKTYGRS | 67 | W | KEEPPQKRL |
| 20 | L | EPRRLVDVF | 44 | P | EPPQKRLPM | 68 | W | LRNLSDPAK |
| 21 | L | FFVPRDSQL | 45 | P | KEDRETDLV | 69 | W | VERRNLEDL |
| 22 | L | LEKTPEDDI | 46 | P | KEEPPQKRL | 70 | W | VKEEPPQKR |
| 23 | L | LILDFRSKL | 47 | P | LRNLSDPAK |  |  |  |
| 24 | L | LPRAAHEIL | 48 | P | RRNLEDLSS |  |  |  |

**Table S5.** 109 B-cell epitopes as well as efficient MHC-II binders

| **Sr. No.** | **Protein** | **Epitopes**  **(B-cell+MHC-II binders)** | **Sr. No.** | **Protein** | **Epitopes**  **(B-cell+MHC-II binders)** | **Sr. No.** | **Protein** | **Epitopes**  **(B-cell+MHC-II binders)** |
| --- | --- | --- | --- | --- | --- | --- | --- | --- |
| 1 | C | LLTLFRRTK | 26 | L | IVSMIEPLV | 51 | L | YYQIDQPFF |
| 2 | C | LTLFRRTKK | 27 | L | LHDEVNDQG | 52 | M | IAAYPLGVG |
| 3 | F | ILHYEKLSK | 28 | L | LIAEQDETV | 53 | M | IFFLSITKL |
| 4 | F | IYKNNTHDL | 29 | L | LILDFRSKL | 54 | M | IRTIAAYPL |
| 5 | F | LEDRRVRPT | 30 | L | LIVDPELFA | 55 | M | VRRAGKYYS |
| 6 | F | LEIYKNNTH | 31 | L | LKKYYQIDQ | 56 | M | YPLGVGKSA |
| 7 | F | LVKGVTRKY | 32 | L | LNLSPLIQR | 57 | M | YYSVDYCRR |
| 8 | F | VEKKRNTYS | 33 | L | LPRAAHEIL | 58 | N | FAPGGYPLL |
| 9 | F | VKGVTRKYK | 34 | L | LQNITRNLI | 59 | N | ILIEVKKGG |
| 10 | F | YVDLSSYYI | 35 | L | LSAYETNTR | 60 | N | LIGGSDQDI |
| 11 | G | IIQNYTRST | 36 | L | LSPLIQRYL | 61 | N | LKTARDSSK |
| 12 | G | IMIIQNYTR | 37 | L | LSQNLLVTS | 62 | N | LRSRLAAKA |
| 13 | G | IQNYTRSTD | 38 | L | LSRYEDNTG | 63 | N | VLIGGSDQD |
| 14 | G | LVVNWRNNT | 39 | L | MDRRVILPR | 64 | N | YPLLWSFAM |
| 15 | G | MIIQNYTRS | 40 | L | MELEKTPED | 65 | P | FTLRNLSDP |
| 16 | G | VLTVNPLVV | 41 | L | METTVKSDI | 66 | P | IQKTYGRSS |
| 17 | L | FEKLSQNLL | 42 | L | MIEPLVLAL | 67 | P | IQQPSIKDQ |
| 18 | L | FFVPRDSQL | 43 | L | MKDKALSPI | 68 | P | IVGISPEDE |
| 19 | L | FHDDLKKYY | 44 | L | VDLARALRS | 69 | P | LEDLSSTSP |
| 20 | L | FLIVDPELF | 45 | L | VMMEDGLLV | 70 | P | LREDLILPE |
| 21 | L | IEGYSQKTW | 46 | L | YLEDEQFNV | 71 | P | LRNLSDPAK |
| 22 | L | IKNSQKPKF | 47 | L | YLHQQDFEK | 72 | P | VERRNLEDL |
| 23 | L | ILPRAAHEI | 48 | L | YMKDKALSP | 73 | P | VIVGISPED |
| 24 | L | IRNNIKNLK | 49 | L | YQPLISRSN | 74 | P | VKEEPPQKR |
| 25 | L | IVPPQKVLE | 50 | L | YSGNLPDSQ | 75 | P | YGLGVKEQN |
| **Sr. No.** | **Protein** | **Epitopes**  **(B-cell+MHC-II binders)** | **Sr. No.** | **Protein** | **Epitopes**  **(B-cell+MHC-II binders)** |  |  |  |
| 76 | P | YGRSSIQQP | 103 | W | VIVGISPED |  |  |  |
| 77 | P | YYGLGVKEQ | 104 | W | VKEEPPQKR |  |  |  |
| 78 | V | FTLRNLSDP | 105 | W | VQPGMFEDH |  |  |  |
| 79 | V | IQKTYGRSS | 106 | W | VVQPGMFED |  |  |  |
| 80 | V | IQQPSIKDQ | 107 | W | YGLGVKEQN |  |  |  |
| 81 | V | ISPEDEEPS | 108 | W | YGRSSIQQP |  |  |  |
| 82 | V | IVGISPEDE | 109 | W | YYGLGVKEQ |  |  |  |
| 83 | V | LEDLSSTSP |  |  |  |  |  |  |
| 84 | V | LRNLSDPAK |  |  |  |  |  |  |
| 85 | V | VERRNLEDL |  |  |  |  |  |  |
| 86 | V | VGISPEDEE |  |  |  |  |  |  |
| 87 | V | VIVGISPED |  |  |  |  |  |  |
| 88 | V | VKEEPPQKR |  |  |  |  |  |  |
| 89 | V | YGLGVKEQN |  |  |  |  |  |  |
| 90 | V | YGRSSIQQP |  |  |  |  |  |  |
| 91 | V | YYGLGVKEQ |  |  |  |  |  |  |
| 92 | W | FEDHPPTKK |  |  |  |  |  |  |
| 93 | W | FTLRNLSDP |  |  |  |  |  |  |
| 94 | W | IQKTYGRSS |  |  |  |  |  |  |
| 95 | W | IQQPSIKDQ |  |  |  |  |  |  |
| 96 | W | ISPEDEEPS |  |  |  |  |  |  |
| 97 | W | IVGISPEDE |  |  |  |  |  |  |
| 98 | W | LEDLSSTSP |  |  |  |  |  |  |
| 99 | W | LRNLSDPAK |  |  |  |  |  |  |
| 100 | W | MFEDHPPTK |  |  |  |  |  |  |
| 101 | W | VERRNLEDL |  |  |  |  |  |  |
| 102 | W | VGISPEDEE |  |  |  |  |  |  |

**Table S6.** List of 16 CTL epitopes as well as MHC-I/II binder

| **Sr. No.** | **Protein** | **CTL Epitopes** | **MHC-I/II binder** |
| --- | --- | --- | --- |
| 1 | C | APVENLNKL | MHC-I binder |
| 2 | C | GECLRMMEM | MHC-I binder |
| 3 | C | KLRGECLRM | MHC-I binder |
| 4 | F | EAMKNADNI | MHC-I binder |
| 5 | G | ILSAFNTVI | MHC-I/II binder |
| 6 | M | FRRNNAIAF | MHC-I/II binder |
| 7 | N | ALNINRGYL | MHC-I binder |
| 8 | N | AYGLRITDM | MHC-I binder |
| 9 | N | NLRSRLAAK | MHC-I binder |
| 10 | V | AVPFTLRNL | MHC-I binder |
| 11 | V | SEDPIIREL | MHC-I binder |
| 12 | W | AVPFTLRNL | MHC-I binder |
| 13 | W | SEDPIIREL | MHC-I binder |
| 14 | L | VSMIEPLVL | MHC-II binder |
| 15 | M | YLKIDADLS | MHC-II binder |
| 16 | P | INSIKLINL | MHC-II binder |

**Table S7.** Catalog of 278 efficient MHC-I and II binders

| **Sr. No.** | **Protein** | **Epitopes** | **Sr. No.** | **Protein** | **Epitopes** | **Sr. No.** | **Protein** | **Epitopes** | **Sr. No.** | **Protein** | **Epitopes** |
| --- | --- | --- | --- | --- | --- | --- | --- | --- | --- | --- | --- |
| 1 | C | FCSAPVENL | 27 | F | FPILTEIQQ | 53 | F | VGILHYEKL | 79 | G | ILSAFNTVI |
| 2 | C | FLKKIGKLI | 28 | F | IASLCIGLI | 54 | F | VKGVTRKYK | 80 | G | IMIIQNYTR |
| 3 | C | IKQKPGKIF | 29 | F | IEIGFCLIT | 55 | F | VLFANCISV | 81 | G | IPSKVIKSY |
| 4 | C | IMEENVPDM | 30 | F | IIVRVYFPI | 56 | F | VLGNVIISL | 82 | G | IRPKLFAVK |
| 5 | C | IYPVLLPQM | 31 | F | IKGALEIYK | 57 | F | VNPSLISML | 83 | G | IRPNSHYIL |
| 6 | C | LMQRCCPML | 32 | F | ILHYEKLSK | 58 | F | VRLAGVIMA | 84 | G | ITIPANIGL |
| 7 | C | LNKLRGECL | 33 | F | ILMISECSV | 59 | F | VRNTLISNI | 85 | G | IYDTGDNVI |
| 8 | C | LQFLKKIGK | 34 | F | IQQAYIQEL | 60 | F | VRPTSSGDL | 86 | G | LLKNKIWCI |
| 9 | C | LQTLYTMIM | 35 | F | ISMLSMIIL | 61 | F | VYFPILTEI | 87 | G | LMMTRLAVK |
| 10 | C | LREGGVITH | 36 | F | ITFISFIIV | 62 | F | YCNLLILIL | 88 | G | LRSIEKGRY |
| 11 | C | LRGECLRMM | 37 | F | IVPNFILVR | 63 | F | YEKLSKIGL | 89 | G | LYFPAVGFL |
| 12 | C | LTLFRRTKK | 38 | F | IVRVYFPIL | 64 | F | YIKEAQRLL | 90 | G | MNIMIIQNY |
| 13 | C | MGMYVLYLM | 39 | F | IYKNNTHDL | 65 | F | YKTRLNGIL | 91 | G | MTRLAVKPK |
| 14 | C | MLPKLQFLK | 40 | F | IYVDLSSYY | 66 | F | YLSDLLFVF | 92 | G | VGLPNNICL |
| 15 | C | MMASILLTL | 41 | F | LDLALSKYL | 67 | F | YYIIVRVYF | 93 | G | VIRPKLFAV |
| 16 | C | MTYNWTQWL | 42 | F | LHYEKLSKI | 68 | G | FAYSHLERI | 94 | G | VNWRNNTVI |
| 17 | C | MYVLYLMQR | 43 | F | LLFVFGPNL | 69 | G | FKDNEILYR | 95 | G | VPSLFMTNV |
| 18 | C | VFNNPASKI | 44 | F | LQDYINTNL | 70 | G | FLIDRINWI | 96 | G | WCISLVEIY |
| 19 | C | VLLPQMELL | 45 | F | LSKYLSDLL | 71 | G | FLLKNKIWC | 97 | G | WSGSLMMTR |
| 20 | C | WLQTLYTMI | 46 | F | LSMIILYVL | 72 | G | FSWDTMIKF | 98 | G | YFAYSHLER |
| 21 | C | YLMQRCCPM | 47 | F | LSSYYIIVR | 73 | G | IALLGSIVI | 99 | G | YGTMDIKKI |
| 22 | C | YPVLLPQME | 48 | F | LVVSSHVPR | 74 | G | IDTSSTITI | 100 | G | YILRSGLLK |
| 23 | C | YRRHTDDQV | 49 | F | LYVLSIASL | 75 | G | IIVMNIMII | 101 | G | YNNEFYYVL |
| 24 | C | YTMIMEENV | 50 | F | MKNADNINK | 76 | G | IKFGDVLTV | 102 | G | YVLCAVSTV |
| 25 | C | YVLYLMQRC | 51 | F | MLSMIILYV | 77 | G | IKKINEGLL | 103 | L | FDPYNMLEY |
| 26 | F | FALSNGVLF | 52 | F | VDLSSYYII | 78 | G | ILRSGLLKY | 104 | L | FFVPRDSQL |
| **Sr. No.** | **Protein** | **Epitopes** | **Sr. No.** | **Protein** | **Epitopes** | **Sr. No.** | **Protein** | **Epitopes** | **Sr. No.** | **Protein** | **Epitopes** |
| 105 | L | FLMDRRVIL | 132 | L | LYLGQSISI | 159 | M | IQLDKHQAL | 186 | N | IEAQIWILI |
| 106 | L | FPISRLFNM | 133 | L | METTVKSDI | 160 | M | IRTIAAYPL | 187 | N | IEQSGRQSV |
| 107 | L | FPLWSTEEL | 134 | L | MGNRIYNIV | 161 | M | ISKRLFAQM | 188 | N | IEVKKGGSA |
| 108 | L | FPVMGNRIY | 135 | L | MMLLYQSTL | 162 | M | IVFGSSGPL | 189 | N | IGPRAPYMV |
| 109 | L | IAYPECNNI | 136 | L | VLEYAPIMK | 163 | M | IYMIPRTML | 190 | N | IKSLMLLYR |
| 110 | L | IAYTPGFPI | 137 | L | VMMEDGLLV | 164 | M | IYTPGANER | 191 | N | IRFGLETRY |
| 111 | L | IFYASLTYL | 138 | L | WRYESMAIF | 165 | M | LDKHQALRI | 192 | N | LETRYPALA |
| 112 | L | IKYQPLISR | 139 | L | YLLCLQKTV | 166 | M | LEELCSLKV | 193 | N | LKTARDSSK |
| 113 | L | IMTESVLQK | 140 | L | YLNLSRIFV | 167 | M | LEFRRNNAI | 194 | N | LLEESIQTK |
| 114 | L | IPFLFLSAY | 141 | L | YPECNNILF | 168 | M | LGSIGGLSL | 195 | N | LLWSFAMGV |
| 115 | L | IQFDCFMEL | 142 | L | YPVLERTRI | 169 | M | LHIKINGVI | 196 | N | LLYREIGPR |
| 116 | L | IRSMFIDDL | 143 | L | YQIDQPFFV | 170 | M | LKIDADLSK | 197 | N | LMRILKTAR |
| 117 | L | LEAMVGRYI | 144 | L | YQQAMLLGL | 171 | M | LQPSIPREF | 198 | N | LRITDMSTL |
| 118 | L | LEGKFRLRL | 145 | L | YVGSSTDER | 172 | M | LTWNNSCEI | 199 | N | LTLFALDVI |
| 119 | L | LERSVIYVA | 146 | L | YYLTPEMVL | 173 | M | MGFQKNLCF | 200 | N | MKVGAAFTL |
| 120 | L | LEVPDALEA | 147 | M | FMIYDDVFI | 174 | M | MLHLGNFVR | 201 | N | MRNLLSQSL |
| 121 | L | LEYAPIMKA | 148 | M | FMLHLGNFV | 175 | M | VFIDNTGRI | 202 | N | MVEILIEVK |
| 122 | L | LILDFRSKL | 149 | M | FNLLVYLKI | 176 | M | VYLKIDADL | 203 | N | MYSERPGAL |
| 123 | L | LPAHASKHI | 150 | M | FQKNLCFSL | 177 | M | YCRRKIDRM | 204 | N | VPATNSPEL |
| 124 | L | LPRAAHEIL | 151 | M | FRRNNAIAF | 178 | M | YMYLICYGF | 205 | N | VQQKRVNPF |
| 125 | L | LPYKVKKEI | 152 | M | FSLGSIGGL | 179 | M | YPLGVGKSA | 206 | N | VRKFMVEIL |
| 126 | L | LQKTVKTIV | 153 | M | FSLMDINPW | 180 | N | FAMGVATTI | 207 | N | WLTEMRNLL |
| 127 | L | LQPKLVSRL | 154 | M | IAFNLLVYL | 181 | N | FAPGGYPLL | 208 | N | YLEPMYFRL |
| 128 | L | LSILNIDNI | 155 | M | IDRMKLQFS | 182 | N | FFALTQQWL | 209 | N | YPALALNEF |
| 129 | L | LSNREVKIW | 156 | M | IFFLSITKL | 183 | N | FFATIRFGL | 210 | N | YPLLWSFAM |
| 130 | L | LTPEMVLMY | 157 | M | IKINGVISK | 184 | N | FMVEILIEV | 211 | P | FAGSSSEVI |
| 131 | L | LVVPLFNGR | 158 | M | INGVISKRL | 185 | N | IDQNMANRL | 212 | P | FEETNASQF |
| **Sr. No.** | **Protein** | **Epitopes** | **Sr. No.** | **Protein** | **Epitopes** | **Sr. No.** | **Protein** | **Epitopes** |  |  |  |
| 213 | P | FFPHDTDRL | 240 | P | YHWSIERSI | 267 | W | LDPVVTDVV |  |  |  |
| 214 | P | FIQKNQKEI | 241 | P | YPSAGTENV | 268 | W | LRNLSDPAK |  |  |  |
| 215 | P | FSFDNVKNF | 242 | P | YQEGKSVNA | 269 | W | LVNDGLNII |  |  |  |
| 216 | P | FTSSPERGW | 243 | V | FAGSSSEVI | 270 | W | MPKSRGIPI |  |  |  |
| 217 | P | IANTVNDII | 244 | V | FIQKNQKEI | 271 | W | VERRNLEDL |  |  |  |
| 218 | P | IKLINLDMR | 245 | V | FTSSPERGW | 272 | W | VGPQTSRNV |  |  |  |
| 219 | P | IPIKKGTDA | 246 | V | ISICWDGKR | 273 | W | VHLENKLST |  |  |  |
| 220 | P | ISPDKTEIV | 247 | V | ISPDKTEIV | 274 | W | VIAEHYYGL |  |  |  |
| 221 | P | LDPVVTDVV | 248 | V | LDPVVTDVV | 275 | W | VKEEPPQKR |  |  |  |
| 222 | P | LEQQSLFSF | 249 | V | LRNLSDPAK | 276 | W | VSDAKMLSY |  |  |  |
| 223 | P | LETLCEESV | 250 | V | LVNDGLNII | 277 | W | YHDHGGECT |  |  |  |
| 224 | P | LIRTHIKDR | 251 | V | MPKSRGIPI | 278 | W | YHWSIERSI |  |  |  |
| 225 | P | LMGVINSIK | 252 | V | VERRNLEDL |  |  |  |  |  |  |
| 226 | P | LRNLSDPAK | 253 | V | VGPQTSRNV |  |  |  |  |  |  |
| 227 | P | LRSELIGYL | 254 | V | VHLENKLST |  |  |  |  |  |  |
| 228 | P | LVNDGLNII | 255 | V | VIAEHYYGL |  |  |  |  |  |  |
| 229 | P | MGVINSIKL | 256 | V | VKEEPPQKR |  |  |  |  |  |  |
| 230 | P | MPKSRGIPI | 257 | V | VSDAKMLSY |  |  |  |  |  |  |
| 231 | P | VGPQTSRNV | 258 | V | WCNPACSRI |  |  |  |  |  |  |
| 232 | P | VHLENKLST | 259 | V | WVEEWCNPA |  |  |  |  |  |  |
| 233 | P | VIAEHYYGL | 260 | V | YHDHGGECT |  |  |  |  |  |  |
| 234 | P | VKEEPPQKR | 261 | V | YHWSIERSI |  |  |  |  |  |  |
| 235 | P | VKNFRDGSL | 262 | W | FAGSSSEVI |  |  |  |  |  |  |
| 236 | P | VLMGVINSI | 263 | W | FIQKNQKEI |  |  |  |  |  |  |
| 237 | P | VQLREDLIL | 264 | W | FTSSPERGW |  |  |  |  |  |  |
| 238 | P | YHADHLGDY | 265 | W | IGKRVSNTR |  |  |  |  |  |  |
| 239 | P | YHDHGGECT | 266 | W | ISPDKTEIV |  |  |  |  |  |  |

| **Table S8.** List of potential siRNAs utilizing the VIRsiRNApred algorithm |
| --- |

| **ID** | **Target Gene** | **Start position** | **End position** | **Sense** | **Antisense** | **Length** | **Inhibition** | **Off-target (seeds)** | **Immunogenicity** |
| --- | --- | --- | --- | --- | --- | --- | --- | --- | --- |
| 19 | N | 689 | 707 | agatgggctaaatacgtcc | ggacgtatttagcccatct | 19 | 0.82 | 12 | Non-immunomodulatory |
| 20 | N | 711 | 729 | aaaagagagtcaatccgtt | aacggattgactctctttt | 19 | 0.88 | 20 | Non-immunomodulatory |
| 21 | N | 712 | 730 | aaagagagtcaatccgttc | gaacggattgactctcttt | 19 | 0.89 | 16 | Non-immunomodulatory |
| 22 | N | 713 | 731 | aagagagtcaatccgttct | agaacggattgactctctt | 19 | 0.9 | 18 | Non-immunomodulatory |
| 23 | N | 714 | 732 | agagagtcaatccgttctt | aagaacggattgactctct | 19 | 0.81 | 13 | Non-immunomodulatory |
| 34 | N | 1272 | 1290 | atcaagttgcagaactcgc | gcgagttctgcaacttgat | 19 | 0.8 | 20 | Immunomodulatory |
| 40 | N | 1677 | 1695 | aaaatgaccttgatttcgt | acgaaatcaaggtcatttt | 19 | 0.83 | 14 | Immunomodulatory |
| 55 | P/V/C | 2473 | 2491 | aaatacagaagacatacgg | ccgtatgtcttctgtattt | 19 | 0.8 | 16 | Immunomodulatory |
| 61 | P/V/C | 2672 | 2690 | aaagagagtgtcgaacacc | ggtgttcgacactctcttt | 19 | 0.87 | 8 | Non-immunomodulatory |
| 71 | P/V/C | 3031 | 3049 | ctgaacactactacggact | agtccgtagtagtgttcag | 19 | 0.81 | 7 | Non-immunomodulatory |
| 80 | P/V/C | 3531 | 3549 | aaaactgagatcgtcaacg | cgttgacgatctcagtttt | 19 | 0.88 | 12 | Non-immunomodulatory |
| 81 | P/V/C | 3532 | 3550 | aaactgagatcgtcaacgg | ccgttgacgatctcagttt | 19 | 0.84 | 19 | Non-immunomodulatory |
| 104 | P/V/C | 3983 | 4001 | aattaatctggatatgcgc | gcgcatatccagattaatt | 19 | 0.82 | 5 | Immunomodulatory |
| 109 | P/V/C | 4080 | 4098 | aagactaacaccgcactct | agagtgcggtgttagtctt | 19 | 0.96 | 19 | Non-immunomodulatory |
| 179 | F | 7192 | 7210 | atactaatttagtaccgac | gtcggtactaaattagtat | 19 | 0.8 | 9 | Non-immunomodulatory |
| 184 | F | 7382 | 7400 | aagaacattgggttacgct | agcgtaacccaatgttctt | 19 | 0.8 | 2 | Non-immunomodulatory |
| 192 | F | 7715 | 7733 | atgtttaacgggatcgact | agtcgatcccgttaaacat | 19 | 0.82 | 17 | Non-immunomodulatory |
| 195 | F | 7778 | 7796 | atttgcactatctaacggg | cccgttagatagtgcaaat | 19 | 0.84 | 14 | Non-immunomodulatory |
| 205 | F | 8075 | 8093 | caaagaggctcaacgactc | gagtcgttgagcctctttg | 19 | 0.8 | 17 | Non-immunomodulatory |
| 206 | F | 8076 | 8094 | aaagaggctcaacgactcc | ggagtcgttgagcctcttt | 19 | 0.81 | 19 | Non-immunomodulatory |
| 220 | G | 8878 | 8896 | aaagtattgttgacgtact | agtacgtcaacaatacttt | 19 | 0.87 | 8 | Non-immunomodulatory |
| 221 | G | 8879 | 8897 | aagtattgttgacgtactg | cagtacgtcaacaatactt | 19 | 0.84 | 20 | Non-immunomodulatory |
| 226 | G | 9016 | 9034 | ttaagagctactacggaac | gttccgtagtagctcttaa | 19 | 0.91 | 14 | Non-immunomodulatory |
| 233 | G | 9330 | 9348 | aagatcagccagtcgactg | cagtcgactggctgatctt | 19 | 0.8 | 10 | Immunomodulatory |
| 237 | G | 9554 | 9572 | atacactttacccgtagtc | gactacgggtaaagtgtat | 19 | 0.87 | 12 | Non-immunomodulatory |
| 244 | G | 9765 | 9783 | aatccaaacaccgtttacc | ggtaaacggtgtttggatt | 19 | 0.83 | 8 | Non-immunomodulatory |
| 256 | G | 9979 | 9997 | ataaagttatgccgtatgg | ccatacggcataactttat | 19 | 0.82 | 8 | Non-immunomodulatory |
| 273 | G | 10784 | 10802 | atataatgataataatcgt | acgattattatcattatat | 19 | 0.8 | 9 | Non-immunomodulatory |
| 283 | G | 11106 | 11124 | ataacaattaatgcgaact | agttcgcattaattgttat | 19 | 0.84 | 11 | Non-immunomodulatory |
| 311 | L | 12683 | 12701 | atctaaacatataatccgt | acggattatatgtttagat | 19 | 0.8 | 15 | Non-immunomodulatory |
| 318 | L | 12976 | 12994 | aatatgtcttatccggtgc | gcaccggataagacatatt | 19 | 0.81 | 15 | Immunomodulatory |
| 338 | L | 13669 | 13687 | attggatgcacaaacgact | agtcgtttgtgcatccaat | 19 | 0.86 | 20 | Immunomodulatory |
| 348 | L | 14441 | 14459 | aaatataggtgatccggtt | aaccggatcacctatattt | 19 | 0.86 | 12 | Non-immunomodulatory |
| 375 | L | 15361 | 15379 | aagcttggtacctagcgtc | gacgctaggtaccaagctt | 19 | 0.88 | 16 | Non-immunomodulatory |
| 378 | L | 15563 | 15581 | aaaggtagatacgaatctt | aagattcgtatctaccttt | 19 | 0.85 | 11 | Non-immunomodulatory |
| 382 | L | 15646 | 15664 | aaactgatgattacaacgg | ccgttgtaatcatcagttt | 19 | 0.88 | 15 | Non-immunomodulatory |
| 390 | L | 15715 | 15733 | atgtaggccaagtagacgc | gcgtctacttggcctacat | 19 | 0.87 | 9 | Immunomodulatory |
| 408 | L | 16487 | 16505 | aaaaatatgtgtactgcgt | acgcagtacacatattttt | 19 | 0.83 | 15 | Non-immunomodulatory |
| 409 | L | 16488 | 16506 | aaaatatgtgtactgcgtg | cacgcagtacacatatttt | 19 | 0.81 | 19 | Non-immunomodulatory |
| 410 | L | 16490 | 16508 | aatatgtgtactgcgtgac | gtcacgcagtacacatatt | 19 | 0.8 | 20 | Non-immunomodulatory |
| 420 | L | 17163 | 17181 | aatttagactcctacgagt | actcgtaggagtctaaatt | 19 | 0.84 | 10 | Non-immunomodulatory |
| 421 | L | 17164 | 17182 | atttagactcctacgagta | tactcgtaggagtctaaat | 19 | 0.8 | 7 | Non-immunomodulatory |
| 432 | L | 17725 | 17743 | aaatcagttatgatatcgg | ccgatatcataactgattt | 19 | 0.8 | 16 | Non-immunomodulatory |

**Table S9.** List of potential siRNAs utilizing the DesiRm algorithm

| **ID** | **Target Gene** | **Start position** | **End position** | **Sense** | **Antisense** | **Length** | **Inhibition** | **Off-target (seeds)** | **Immunogenicity** |
| --- | --- | --- | --- | --- | --- | --- | --- | --- | --- |
| 4 | N | 917 | 935 | gcaaccatcagattcgggt | acccgaatctgatggttgc | 19 | 56.33 | 13 | Non-immunomodulatory |
| 12 | N | 2199 | 2217 | ctaccaggtaatgctcgca | tgcgagcattacctggtag | 19 | 55.54 | 19 | Non-immunomodulatory |
| 14 | P/V/C | 2667 | 2685 | attggaaagagagtgtcga | tcgacactctctttccaat | 19 | 57.87 | 8 | Non-immunomodulatory |
| 15 | P/V/C | 2668 | 2686 | ttggaaagagagtgtcgaa | ttcgacactctctttccaa | 19 | 57.05 | 7 | Non-immunomodulatory |
| 16 | P/V/C | 2670 | 2688 | ggaaagagagtgtcgaaca | tgttcgacactctctttcc | 19 | 56.76 | 11 | Non-immunomodulatory |
| 35 | P/V/C | 3724 | 3742 | gcaagagtgtcaatgcgga | tccgcattgacactcttgc | 19 | 65.32 | 18 | Non-immunomodulatory |
| 36 | P/V/C | 3725 | 3743 | caagagtgtcaatgcggag | ctccgcattgacactcttg | 19 | 56.35 | 9 | Non-immunomodulatory |
| 38 | P/V/C | 3757 | 3775 | atgcttccactgcggttaa | ttaaccgcagtggaagcat | 19 | 55.63 | 2 | Non-immunomodulatory |
| 62 | G | 9938 | 9956 | acatcaacttgccctacga | tcgtagggcaagttgatgt | 19 | 56.52 | 14 | Non-immunomodulatory |
| 80 | L | 13056 | 13074 | gctggacgattgttcgcaa | ttgcgaacaatcgtccagc | 19 | 55.43 | 2 | Non-immunomodulatory |
| 82 | L | 13059 | 13077 | ggacgattgttcgcaaaga | tctttgcgaacaatcgtcc | 19 | 55.2 | 14 | Non-immunomodulatory |
| 86 | L | 13591 | 13609 | gtctcaattggagatacga | tcgtatctccaattgagac | 19 | 55.81 | 12 | Immunomodulatory |
| 89 | L | 13670 | 13688 | ttggatgcacaaacgacta | tagtcgtttgtgcatccaa | 19 | 55.1 | 17 | Immunomodulatory |
| 90 | L | 13672 | 13690 | ggatgcacaaacgactaga | tctagtcgtttgtgcatcc | 19 | 58.62 | 7 | Non-immunomodulatory |
| 97 | L | 15018 | 15036 | atgtggagggaattagcgt | acgctaattccctccacat | 19 | 56.08 | 8 | Non-immunomodulatory |
| 104 | L | 15298 | 15316 | ctgctatcagaattgcgac | gtcgcaattctgatagcag | 19 | 55.05 | 8 | Immunomodulatory |
| 108 | L | 15903 | 15921 | actgttgctcagaccgttc | gaacggtctgagcaacagt | 19 | 56.81 | 16 | Non-immunomodulatory |
| 110 | L | 15905 | 15923 | tgttgctcagaccgttctt | aagaacggtctgagcaaca | 19 | 55.5 | 13 | Non-immunomodulatory |
